# Canine polarized macrophages express distinct functional and transcriptomic profiles

**DOI:** 10.3389/fvets.2022.988981

**Published:** 2022-11-01

**Authors:** Lyndah Chow, Sirikul Soontararak, William Wheat, Dylan Ammons, Steven Dow

**Affiliations:** ^1^Flint Animal Cancer Center, Department of Clinical Sciences, College of Veterinary Medicine and Biomedical Sciences, Colorado State University, Ft. Collins, CO, United States; ^2^Department of Companion Animal Clinical Sciences, Faculty of Veterinary Medicine, Kasetsart University, Bangkok, Thailand

**Keywords:** RNA sequencing, transcriptome, dog, cytokines, macrophage, macrophage phenotype and function

## Abstract

Macrophage differentiation and function in disease states is highly regulated by the local microenvironment. For example, macrophage exposure to IFN-γ (interferon gamma) initiates the development of inflammatory (M1) macrophages, which acquire anti-tumoral and antimicrobial activity, while exposure to IL-4 (interleukin-4) and IL-13 (interleukin-13) drives an anti-inflammatory (M2) macrophage phenotype, which promotes healing and suppression of inflammatory responses. Previous studies of canine polarized macrophages have identified several surface markers that distinguished GM-CSF (granulocyte macrophage colony stimulating factor), IFN-γ and LPS (lipopolysaccharide) derived M1 macrophages or M2 macrophages; and reported a subset of genes that can be used to differentiate between polarization states. However, the need remains to understand the underlying biological mechanisms governing canine macrophage polarization states. Therefore, in the present study we used transcriptome sequencing, a larger panel of flow cytometry markers, and the addition of antimicrobial functional assays to further characterize canine macrophage polarization. Transcriptome analysis revealed unique, previously unreported signatures and pathways for polarized canine M1 and M2 macrophages. New flow cytometric markers were also identified, along with new characterization of how macrophage polarization impacted antimicrobial functions. Taken together, the findings reported here provide new insights into canine macrophage biology and identify new tools for the evaluation of polarized macrophages in dogs.

## Introduction

Macrophages play a critical role in cancer immunity, inflammation, healing, elimination of pathogens, and presentation of antigens to T cells. These antigen presenting cells alter their phenotype and functional state based on cues from their local tumor microenvironment (including stromal and tumor cells, extracellular matrix proteins, and pattern recognition receptor ligands). Traditionally, macrophage classification has been dichotomized into two main polarization states: inflammatory (M1) macrophages or anti-inflammatory (M2) macrophages. However, it is now recognized that this categorical classification system does not capture the biological subtleties. As such, a spectrum of macrophage functional states is now regarded as a more biologically relevant classification scheme (1–6). Despite this, the M1 and M2 dichotomy still provides a valuable framework for evaluating macrophage function, especially in large animal disease animal models. The work reported here describes transcriptomic and phenotypic responses of canine macrophages when exposed to inflammatory (IFN-γ) or anti-inflammatory (IL-4 and IL-13) cytokines with comparison to resting (M0) macrophages cultured in M-CSF (macrophage colony-stimulating factor); and defines a group of genes that can be used to characterize macrophage polarization in altered immune environments and make key contributions to bridge the gap between canine and human macrophage biology to enhance the translational value of the dog cancer model.

When macrophages are exposed to IFN-γ, a series of stereotypic changes occur which result in an inflammatory macrophage phenotype. These changes include: changes to surface protein expression, upregulation of anti-tumor and anti-microbial activity, secretion of inflammatory cytokines, and enhanced antigen presentation processes (2, 3, 7–11). In contrast, exposure to IL-4 and IL-13 promote the differentiation of macrophages toward anti-inflammatory phenotype. This cytokine milieu induces upregulation of STAT6 mediated signaling, secretion of anti-inflammatory cytokines (IL-10), and stromal remolding cytokines (TGF-β, VEGF and FGF) (6, 12).

The distribution and polarization state of macrophage in canine tissues is well documented, particularly in tumor tissues (13–17). In one recent study, *in vitro* generated canine M1 macrophages were reported to upregulate expression of iNOS, while M2 macrophages exhibited upregulated expression of the mannose receptor CD206 (18). Other studies have used immunochemistry to assess macrophage numbers and phenotypes in dogs with inflammatory bowel diseases, chronic leishmaniasis (19) and in several different types of cancer, including osteosarcoma (13), mammary tumors (14, 20) and melanoma (21).

Transcriptomic analysis using next generation sequencing provides a powerful tool to complete an in-depth investigation of the cellular processes that characterize macrophages undergoing polarization. An earlier study of *in vitro* polarized canine macrophages used microarray analysis and revealed important gene expression correlates with canine M1 and M2 macrophages (18). However, mRNA sequencing offers several important advantages over microarray studies, including measurement of all messenger RNA (including low abundance transcripts), and more sensitive differential gene expression (DEG) analysis with a wider dynamic range than that of microarray (22).

In the present study, we describe canine macrophage polarization states through use of RNA sequencing and screening of a broader array of antibodies for recognition of surface and intracellular proteins. The findings reported here provide new insights into how canine macrophages respond to their environment cues and regulate chronic infections and cancer.

## Materials and methods

### Generation of monocyte-derived macrophages

Peripheral blood mononuclear cells (PBMC) were purified from EDTA blood samples obtained from healthy female spayed beagles (approximately 2 years old), using Ficoll density gradient separation (Sigma-Aldrich, St. Louis, MO), as described previously (23). The study and use of blood samples was reviewed and approved by the Colorado State University Institutional Animal Care and Use Committee. After Ficoll separation, PBMC were plated in 24-well plates (Corning Inc, Corning NY) at a concentration of 4 × 10^6^ cells per mL in complete culture medium, which consisted of high-glucose DMEM (Thermo Fisher Scientific, Waltham MA) supplemented with 10% FBS (Peak Serum Inc, Wellington CO), non-essential amino acids, essential amino acids, glutamine, and penicillin-streptomycin solution (Sigma-Aldrich, St. louis MO). For monocyte adherence, PBMC were placed in culture for 4 h with 2% FBS media, after which non-adherent cells were removed by aspiration after gently swirling the plates, and the remaining adherent cells were re-cultured in complete medium. The adherent monocytes were then differentiated for 7 days in complete medium, supplemented with 10 ng/mL recombinant human M-CSF (PeproTech, Rocky Hill NJ) to induce a “resting” macrophage (M0) phenotype. Culture medium and M-CSF were replaced every 3 days.

### Macrophage polarization

After 7 days in culture, cytokines were added to the complete culture medium to induce macrophage polarization. To induce M1, inflammatory macrophages, M0 (resting macrophages) were treated with 20 ng/mL of canine IFN-γ (R&D Systems Inc., Minneapolis, MN), while cells were treated with 20 ng/mL each of canine IL-13 and IL-4 (R&D Systems Inc., Minneapolis, MN) to induce M2, anti-inflammatory macrophages.

### Flow cytometry

Macrophages for flow cytometric analysis were detached from culture plates using Accumax cell detachment solution (StemCell Technologies, Vancouver BC). After detachment, cells were resuspended in flow cytometry buffer for PBS with 2% FBS for immunostaining. For intracellular staining, cells were permeabilized using saponin (0.15% in PBS) (Sigma-Aldrich, St. Louis, MO) after fixation with 4% Paraformaldehyde (Therno Fisher, Waltham MA). Primary antibodies used in the study included the following: rabbit polyclonal anti-human suppressor of cytokine signaling 1 (SOCS-1) (Bio-Rad, Hercules CA), rabbit polyclonal anti-mouse iNOS (#PA3-030A, Thermo Fisher, Waltham MA), mouse monoclonal anti-human arginase I (Arg1, clone 19) (BD Biosciences, San Jose, CA), rabbit polyclonal anti- mouse tissue transglutaminase 2 (TGM2) (Bio-Rad), mouse anti-human CD206-PE (macrophage mannose receptor) (Clone 3.29B1.10) (Beckman Coulter, Brea CA), rabbit polyclonal anti-mouse resistin-like molecule α (RELMα) (PeproTech, Rocky Hill NJ), sheep polyclonal anti-human indoleamine-pyrrole 2,3 dioxygenase (IDO) (Bio-Rad, Hercules, CA) and rabbit polyclonal CXCL10 (IP-10) (BioRad). Negative controls included cells that were labeled with isotype-matched, irrelevant target antibodies, including mouse IgG1 (Thermo Fisher) or ChromPure Rabbit IgG (Jackson Immuno Research Labs, West Grove PA).

After staining with appropriately diluted primary antibodies (or isotype matched, irrelevant antibodies), cells were washed and then labeled with secondary antibodies, which included AffiniPure donkey anti-rabbit or anti-mouse IgG (Jackson ImmunoResearch Labs, West Grove, PA). Immunolabeled cells were analyzed on a Beckman Coulter Gallios flow cytometer (Brea, CA), and the flow cytometry data were then analyzed using FlowJo Software v10.5 (Ashland, OR).

### Macrophage immunocytochemistry

Macrophages were grown in 8-well chamber slides for 7 days, using culture conditions described above, and then fixed with 4% paraformaldehyde (Fisher Scientific, Hampton NH) for 10 min at room temperature, then washed twice with PBS. Next, the cells were permeabilized using 0.1% Triton X in PBS for 15 min at room temperature and then blocked with 10% normal donkey serum in 1% BSA for 1 h (Jackson Immuno Research Labs, West Grove PA). Primary antibodies (or irrelevant isotype matched antibodies) as described for flow cytometry (above) were then added and incubated overnight at 4°C with 0.25% saponin for permeabilization. Following overnight incubation with primary antibodies, the slides were washed and incubated with appropriate secondary antibodies in 0.25% saponin in PBS for 1 h, washed twice, followed by addition of DAPI (diamidino-2-phenylindole) for nuclear staining (Thermo Fisher, Waltham, MA). Slides were cover slipped and mounted with ProLong™ Diamond Antifade Mounting medium (Thermo Fisher, Waltham, MA) and were then examined under fluorescence microscopy using an Olympus IX83 spinning disk confocal microscope. A series of 10 random fields per slide were imaged at 20X magnification, and the images were than subject to image quantification, using ImageJ software, and the median fluorescence intensity for marker expression was calculated. Background fluorescence threshold for each channel was set based on matched negative isotype control staining. Examples of negative isotype staining is included in Supplementary Figure S1.

### Expression of co-stimulatory molecules by polarized macrophages

Polarized macrophages were generated as noted above, and then immunolabeled with the following co-stimulatory molecule antibodies: PE-conjugated rat anti-human CD86 (Clone IT2.2; eBioscience, San Diego, CA); FITC-conjugated rat anti-canine MHCII (Clone YKIX334.2; Bio-Rad, Hercules, CA); or Alexa Fluor^®^ 647-conjugated anti-human CD40 (Clone LOB7/6; Bio-Rad, Hercules, CA). Immunolabeled cells were analyzed on a Beckman Coulter Gallios flow cytometer (Brea, CA), and data were analyzed using FlowJo Software.

### Cytokine secretion by polarized macrophages

Supernatants from polarized and control macrophages (triplicate cultures for each condition) were collected after 24 h in culture stimulated with 600 ng/mL of LPS (Sigma-Aldrich, St. louis, MO), and assessed for release of MCP-1, IL-8, IL-10, TNF-α, and IL-1β using canine specific DuoSet ELISAs (R&D Systems Inc., Minneapolis, MN). All ELISAs were performed according to manufacturer recommended protocols. In some studies, macrophages were also stimulated with LPS (10 ng/mL) to generate an internal positive control for cytokine release.

### Phagocytosis assay

Macrophage phagocytosis was quantitated using a 6 h assay, as previously described (24). Briefly, the assay used log phase *S. aureus* stained with a phHrodo Red Phagocytosis Particle Labeling kit (Thermo Fisher, Walthamh MA), in accordance with manufacturer instructions. Labeled *S. aureus* was added to macrophage cultures at an MOI of 5 (5 bacteria per macrophage). Cells with bacteria were then cultured in an IncuCyte instrument (Essen BioScience Inc), and images (9 images per well) were collected every 15 min using a 10X objective, and the average red object integrated intensity was analyzed using IncuCyte S3 Software.

### Measurement of NO2^−^ production

Nitric oxide release was measured using the Griess reagent and macrophage supernatants after 48 h stimulation with 600 ng/mL of LPS, according to manufacturer protocols (Promega, Madison, WI).

### Bactericidal assay

Macrophage bactericidal activity was assessed as described previously (23, 25). Briefly, cultures of *S. pseudointermedius* were propagated in antibiotic-free macrophage growth medium overnight and grown to log phase prior to addition to macrophage cultures. Macrophages were infected with *S. pseudointermedius* at an MOI of 3 in 100 uL of pre-warmed HBSS (Hanks' Balanced Salt Solution) (Thermo Fisher, Waltham, MA) containing Ca^++^ and Mg^++^ and 10% normal, heat-inactivated dog serum, then incubated for 1 h to allow phagocytosis of bacteria. After 1 h of incubation, extracellular bacteria were removed by washing and infected macrophages were either lysed immediately (T0) or cultured for 3 additional hours (T3hr) to assess intracellular killing activity. Numbers of viable bacteria were determined by plating serial 10X dilutions of macrophage lysates on LB agar in 4-quadrant plates (Thermo Fisher Scientific). Manual determination of CFU was done after 24 h of culture at 37°C. Bacterial colony counts at time 0 and after 3 h of culture were determined and the differences in CFU between 0 and 3 h (T3hr-T0) were expressed as the mean percent change in CFU, which was used as a measure of bactericidal activity.

### RNA sequencing

mRNA sequencing was performed on cultured macrophages as previously described (26). Briefly, macrophages were incubated in polarizing cytokines for 24 h prior to RNA extraction. RNA was extracted using a RNeasy mini kit (Qiagen, Hilden Germany), and the RNA was then subjected to sequencing at Novogene Corp, using an Illumina platform (Novogene Co., Sacramento, CA), as described previously (24, 27, 28). RNA samples were tested for RNA quality using an Agilent 2100 Bioanalyzer system, and RNA integrity numbers for macrophage samples ranged from 8.5 to 9.4.

Libraries were sequenced on an Illumina PE150 (HiSeq) platform with a 250 to 300 bp insert cDNA library, for 40M raw reads per sample. Raw data were filtered by removing reads containing adapters and reads containing *N* > 10% and for Phred scores >30. The filtered reads obtained from Novogene were analyzed using Partek Flow software, version 7.0. Filtered reads were aligned with STAR 2.7.3a, CanFam3.1 genome assembly. Aligned reads were annotated and counted using HT-seq (29), and differentially expressed genes were identified using DEseq2 (30). Further biological interpretations including gene ontology enrichment and gene set enrichment analysis were then performed using GSEA (https://www.gsea-msigdb.org/gsea/index.jsp).

The data discussed in this publication have been deposited in NCBI's Gene Expression Omnibus and are accessible through GEO Series accession number GSE207661 (https://www.ncbi.nlm.nih.gov/geo/query/acc.cgi?acc=GSE207661).

### Statistical analyses

Statistical analyses were done using Prism8 software (GraphPad) and the results are shown as the mean ± SD (unless otherwise stated), with the significance set at *p* < 0.05. The normality of the data was examined using the Shapiro-Wilk normality test. Comparisons between 3 or more groups were done using a one-way ANOVA, followed by Tukey's multiple comparisons *post-hoc* test, or as otherwise stated. Most experiments were repeated 3 times, using macrophages generated from different, unrelated dogs. DEG calculations for RNA seq were computed with DEseq2, significance denoted as *p*-value with false discovery rate (FDR) ≤ 0.05.

## Results

### Identification of flow cytometric markers to define polarization states

**Figure 1:**
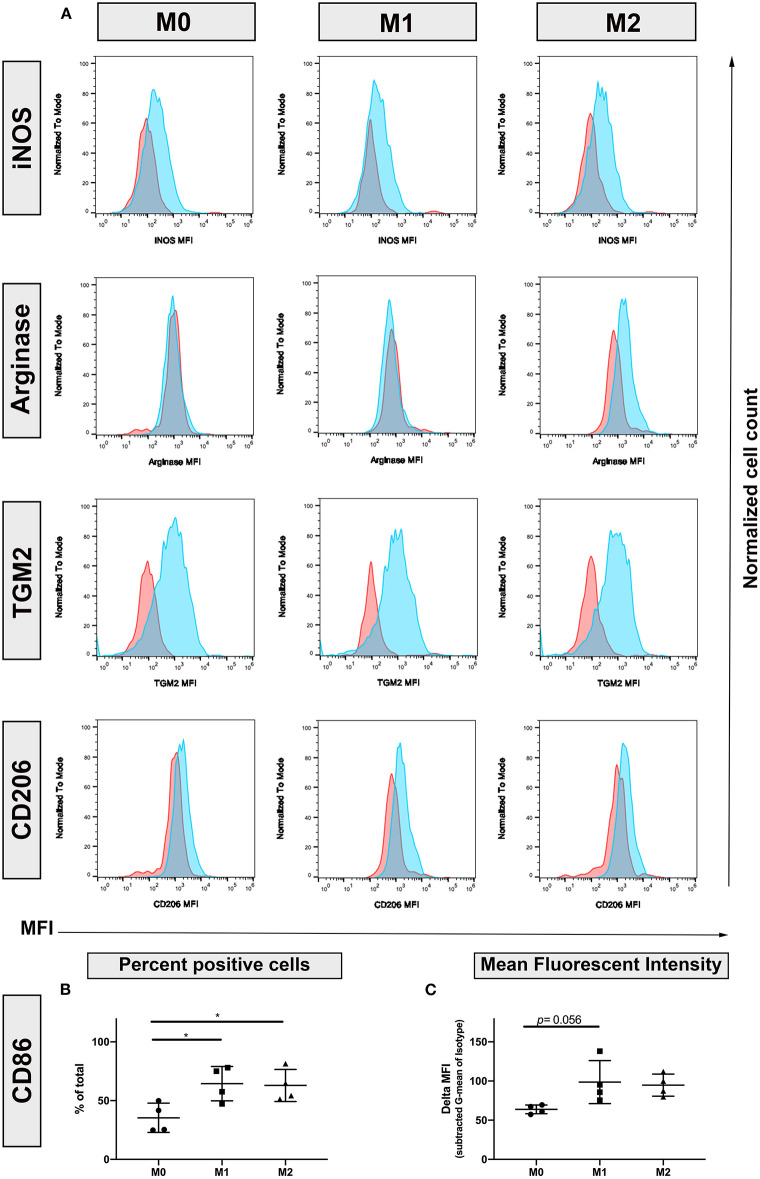
Identification of canine macrophage polarization by flow cytometry. Monocyte-derived macrophages were generated from blood of healthy dogs by *in vitro* culture with or without cytokines as described in methods, and expression of molecules associated with M1 and M2 macrophages was assessed using flow cytometry and cross-reactive antibodies iNOS, inducible Nitric Oxide Synthase; Arginase, TGM2, Transglutaminase 2; CD206, macrophage mannose receptor. **(A)** Primary antibody staining depicted in blue histograms, while isotype matched, irrelevant antibody staining depicted by red histograms. Marker abbreviation: M0, unstimulated macrophages; M1, macrophages activated with IFN-γ; M2, macrophages activated with IL-4 and IL-13; **(B)** CD86 expression. % positive cells of total cells immunostained **(C)** MFI, mean fluorescence intensity. Normalized to isotype controls.

To better define the phenotypic markers associated with polarization in canine macrophages, we screened a panel of antibodies that are known to be upregulated through polarization of human and murine macrophages (13, 14, 16, 18, 21, 28, 31). Screened antibodies included those recognizing Arg1, iNOS, CXCL10, IDO, SOCS-1, Fizz1/RELMα, CD206, and TGM2. The antibodies were initially evaluated for recognition of both surface and intracellular expressed molecules, using flow cytometry (Figure 1). This screen identified 4 cross-reactive antibodies, including intracellular iNOS (upregulated by M1 macrophages), arginase and TGM2. Figure 1A as well as CD206 (surface). The Fizz1/RELMα, CXCL10 and IDO, used in these experiments were negative or not cross-reactive.

We next evaluated the impact of polarization on expression of 3 key macrophage co-stimulatory molecules (MHCII, CD86, and CD40). Both IFN-γ and IL-4/IL-13 polarized macrophages exhibited upregulated expression of CD86 (Figures 1B,C), compared to resting (M0) macrophages. Unexpectedly, macrophage expression of MHCII and CD40 was not upregulated by M1 polarization (data not shown).

### Intracellular expression of macrophage polarization markers

**Figure 2:**
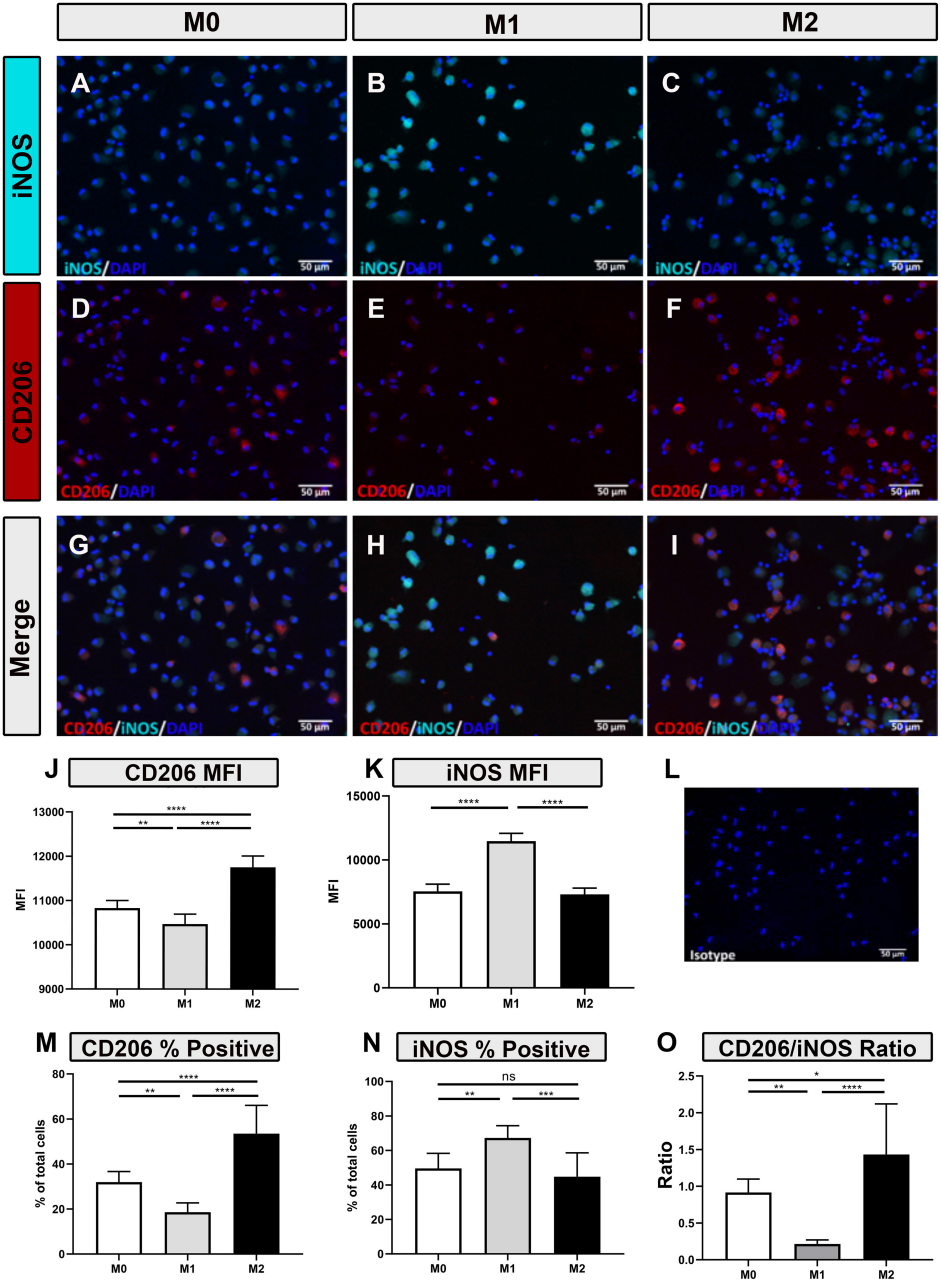
Macrophage responses to polarization as assessed by immunofluorescence staining for iNOS and CD206 intracellular expression. **(A–C)** Immunoflourescence (IF) staining of iNOS expression (cyan) **(D–F)** CD206 (red) in M0, M1 and M2 macrophages as described in Methods, DAPI nuclear staining in blue **(G–I)** Merged images of immunolabeled M0/M1/M2 macrophage (left, middle, and right columns, respectively). Immunostaining with isotype control antibody is shown in image **(L)**. Graphical representation of Mean Fluorescent Intensity (MFI) of CD206 expression **(J)** and iNOS expression **(K)**, and the **(M)** percentage of cells expressing CD206 or **(N)** percentage expressing iNOS **(O)**. Ratio of MFI by CD206 expressed cells (numerator) and iNOS expressing cells (denominator), was calculated for M0, M1, and M2 macrophages. Scale bar indicates 50 μm. Values are plotted as mean ± SD. Statistical differences assessed using One-way ANOVA, followed by Tukey's multiple means adjustment (*p ≤ 0.05, **p ≤ 0.01, ***p ≤ 0.001, **** *p* ≤ 0.0001).

**Figure 3:**
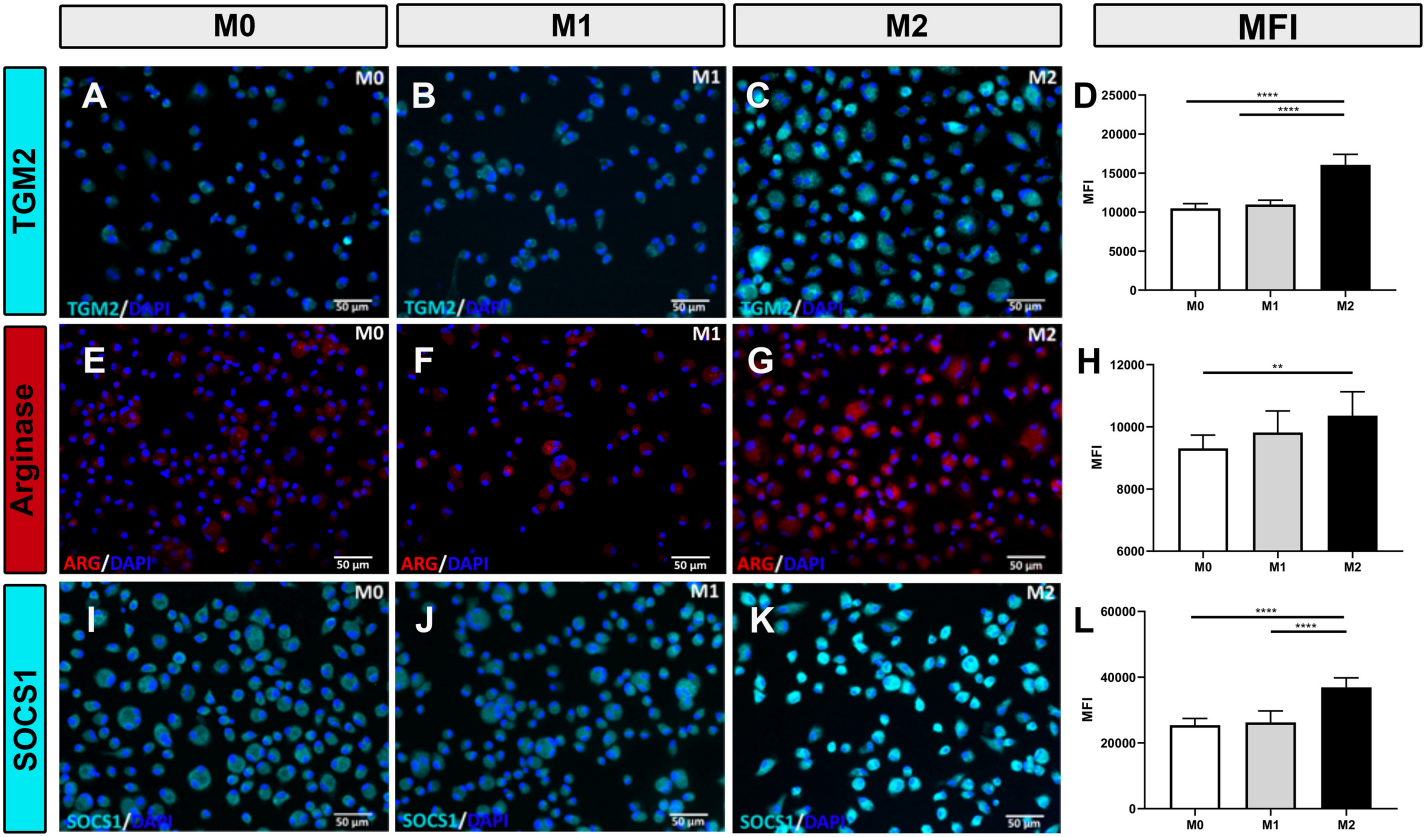
Macrophage responses to polarization assessed by immunofluorescence staining for intracellular TGM2, arginase, or SOCS1 expression. **(A–C)** Immunofluorescence staining for TGM2 expression (cyan) by M0, M1, and M2 differentiated macrophages (left, middle, and right columns, respectively. **(D)** Mean fluorescence intensity (MFI) for intracellular TGM2 expression depicted in bar graphs. **(E–G)** Immunofluorescence staining for intracellular expression of arginase (red). MFI for arginase expression depicted in bar graph **(H)**. **(I–K)** Immunofluorescence staining for intracellular expression of SOCS1 (cyan). MFI for SOCS1 expression depicted in bar graph. **(L)** Values are plotted as mean ± SD. Statistical differences assessed using One-way ANOVA, followed by Tukey's multiple means adjustment (*p ≤ 0.05, **p ≤ 0.01, ***p ≤ 0.001, **** *p* ≤ 0.0001). Scale bar indicates 50 μm.

Polarized macrophages were also screened for intracellular expression of relevant immune molecules using immunofluorescence staining. Intracellular antibodies that were found to be cross-reactive with immune-related proteins expressed by canine macrophages include iNOS (Figures 2A–C), CD206 (Figures 2D–F), TGM2 (Figures 3A–D), arginase (Figures 3E–H) and SOCS1 (Figures 3I–L). We found that IFN-γ polarized, inflammatory M1 significantly upregulated intracellular expression of iNOS (Figures 2B,H,K,N), consistent with previous reports (3, 32–34). Under IL-4/IL-13 polarizing conditions, M2 macrophages significantly upregulated expression of CD206 (Figures 2I,J,M), arginase (Figures 3G,H), TGM2 (Figure 3C), and SOCS1 (Figure 3K). Upregulated expression of CD206 by M2 macrophages was noted in an earlier report (35), but the other 3 markers (arginase, TGM2, and SOCS1) have not been previously reported as to expression by canine M2 macrophages. Furthermore, CD206 and iNOS expression were found to be most accurate in discriminating between M1, M2 and M0 phenotypes (Figures 2G,H,I); with significant differences between total percentage of positive cells expressing the markers, along with differences in total cellular expression, as assessed by mean fluorescence intensity (Figures 2J–N). We next evaluated the utility of using ratios of the above marker expression levels to help further discriminate the 3 polarization states in canine macrophages. From this screen, the ratio of CD206 to iNOS expression provided significant and clear distinction between the 3 polarization states of canine macrophages (Figure 2O). In summary, these studies identified a panel of cross-reactive antibodies that could be used to define polarized canine macrophages in tissues and provide better discrimination of the different macrophage polarization states.

### Polarized macrophages display unique inflammatory and bactericidal activities

**Figure 4:**
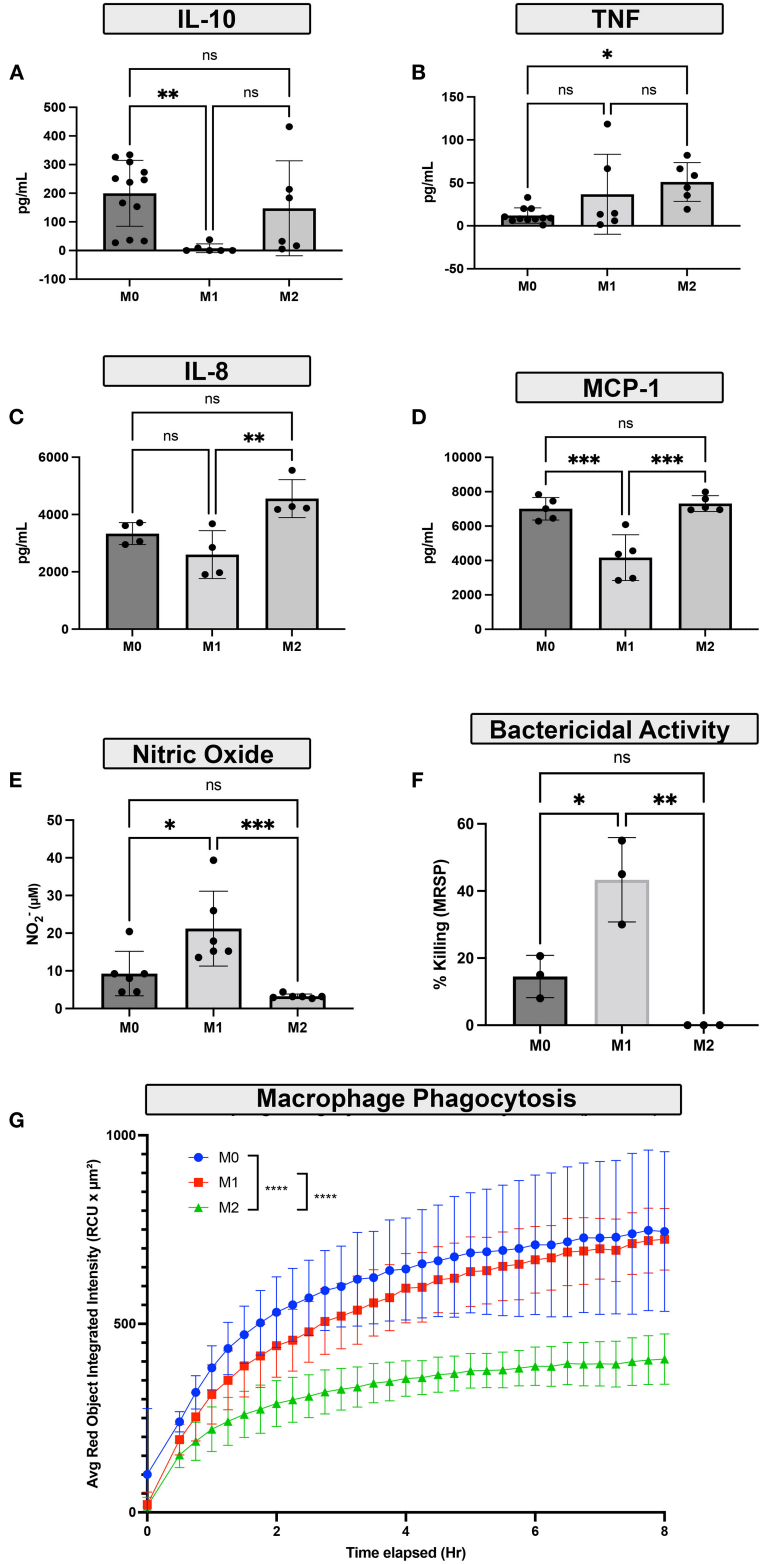
Impact of polarization on macrophage functional responses. **(A–D)** Monocyte-derived macrophages were polarized by cytokine culture to either M1, M2 polarization states, or resting (M0). The LPS stimulated cytokine responses of polarized macrophages over 48h in culture following polarization were assessed using cytokine ELISA. **(A)** IL-10 release **(B)** TNF-α release **(C)** IL-8 release **(D)** MCP-1. **(E)** Nitric oxide (NO) production in response to LPS stimulation was measured by Griess reaction assay. **(F)** The impact of macrophage polarization on bactericidal activity was assessed by killing of *S. pseudointermedius*, bactericidal assay was performed with 1 h for phagocytosis followed by 3 h of bacterial killing activity. Bar graph shows percent killing as ratio of 3-h bacterial growth compared to baseline, as colonies in CFU/mL. **(G)** The effects of polarization on bacterial phagocytosis were measured over time using labeled *S. aureus* and quantified by Incucyte instrument. For the phagocytosis assay, M0 macrophages are depicted in blue, M1 macrophages were depicted in red, and M2 macrophages were depicted in green. The x-axis denotes incubation time in hours. Data are depicted graphically as mean ± SD. Statistical differences were determined using one-way ANOVA, followed by Tukey's multiple comparisons adjustment (*p ≤ 0.05, **p ≤ 0.01, ***p ≤ 0.001, **** *p* ≤ 0.0001), ns for non-significant.

The next studies focused on defining the functional properties of polarized canine macrophages. Basal cytokine secretion was assessed first, using a panel of 5 cytokines relevant to macrophage biology. Neither of the 3 macrophage populations exhibited significant differences in basal cytokine secretion, and total concentrations were low (data not shown). We then evaluated cytokine production by LPS-activated macrophages. Under these conditions, we observed that M1 polarized macrophages exhibited significantly downregulated secretion of MCP-1 and IL-10 (Figures 4A,D) compared to M0 or M2 macrophages. In contrast, M2 macrophages secreted significantly more IL-8 (Figure 4C) compared to M1 macrophages, and also significantly more TNF-α (Figure 4B) compared to resting M0 macrophages. Despite a trend toward increased IL-1β production in the M1 macrophages, the concentrations did not reach statistical significance when compared to either M0 or M2 macrophages (data not shown).

The impact of polarization on the ability of macrophages to phagocytose and kill relevant intracellular bacteria was assessed next, using *S. aureus* and *S. pseudointermedius* as target bacteria M2 macrophages were significantly less effective in phagocytosing *S. aureus* than either M0 or M2 macrophages (Figure 4G) and were also significantly less able to kill intracellular *S. pseudointermedius* than either M0 or M1 macrophages (Figure 4F). Production of NO (Nitric oxide), a key molecule responsible for bactericidal activity, was also significantly depressed in M2 macrophages (Figure 4E), while M1 macrophages produced more NO and generated significantly increased bactericidal activity.

### Transcriptomic profile of polarized macrophages identifies specific gene signatures in dogs

**Figure 5:**
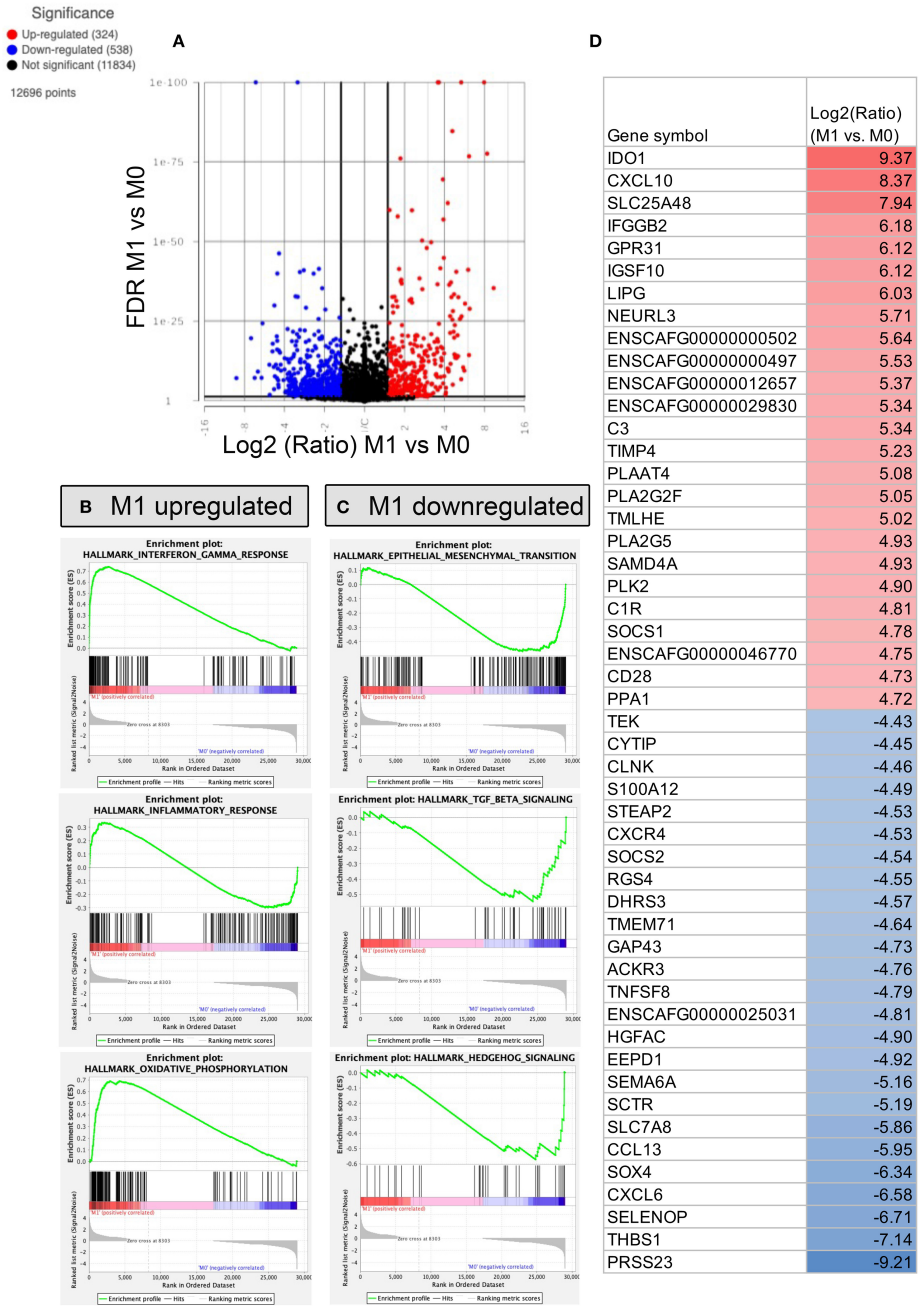
RNA sequencing and transcriptomic analysis of canine polarized macrophages, with comparison of M1 to M0 macrophages. To assess macrophage transcriptome responses to polarization, cells were cultured for 24 h in IFN-γ (M1 macrophages), or no added cytokine (M0), then RNA was sent for sequencing on an Illumina Hiseq sequencer. **(A)** A volcano plot is used to depict differentially expressed genes, with x-axis cutoff (black line) at 1.5 Log2 fold change, y-axis cut off at FDR (false discovery rate) 0.05. Upregulated genes are depicted in red, downregulated genes in blue. **(B)** Gene Set Enrichment Analysis (GSEA) Hallmarks pathway analysis was performed, using normalized counts of M1 and M0 macrophages. The top 3 most upregulated pathways in M1 macrophages are displayed as GSEA plots. The top graph with green peak depicts the enrichment score, followed by black lines indicating where the members of the gene set appear in the ranked list of genes. The bottom portion of the graph shows the value of the ranking metric indicating gene correlations with pathways. **(C)** GSEA Hallmarks pathway analysis of top 3 pathways enriched in M0 macrophages, with similar figures as for B. **(D)** Table showing the top 25 most upregulated and downregulated genes comparing M1 vs. M0 polarized macrophages. The Log2 fold upregulated or downregulated gene expression is depicted, with upregulated genes in red, and most downregulated genes depicted in blue.

**Figure 6:**
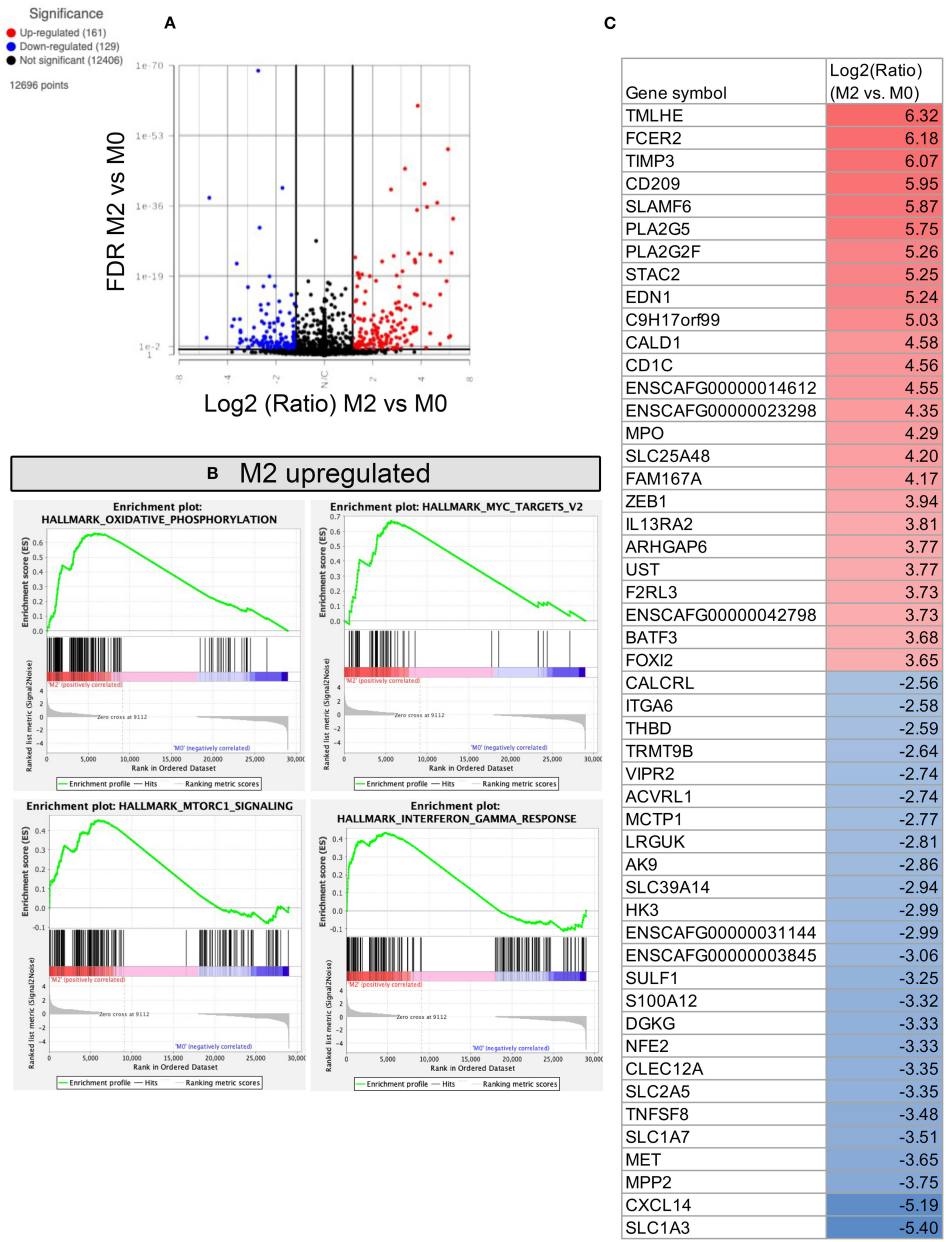
RNA sequencing and transcriptomic analysis of canine polarized macrophages, with comparison of M2 to M0 macrophages. **(A)** A volcano plot depicting differentially expressed genes, with x-axis cut off (black line) at 1.5 Log2 fold change, y-axis cut off at FDR (false discovery rate) 0.05. Upregulated genes are in red, downregulated genes in blue. **(B)** GSEA Hallmarks pathway analysis, using normalized counts generated from M2 and M0 macrophages. The top 4 significantly upregulated pathways in M2 are displayed as GSEA plots. No downregulated pathways were statistically significant. **(C)** Table depicting the top 25 most upregulated and downregulated genes comparing M2 vs. M0 polarized macrophages. Figure lists Log2 fold change values, with highest fold change upregulated genes in red, and lowest fold change downregulated genes in blue.

**Figure 7:**
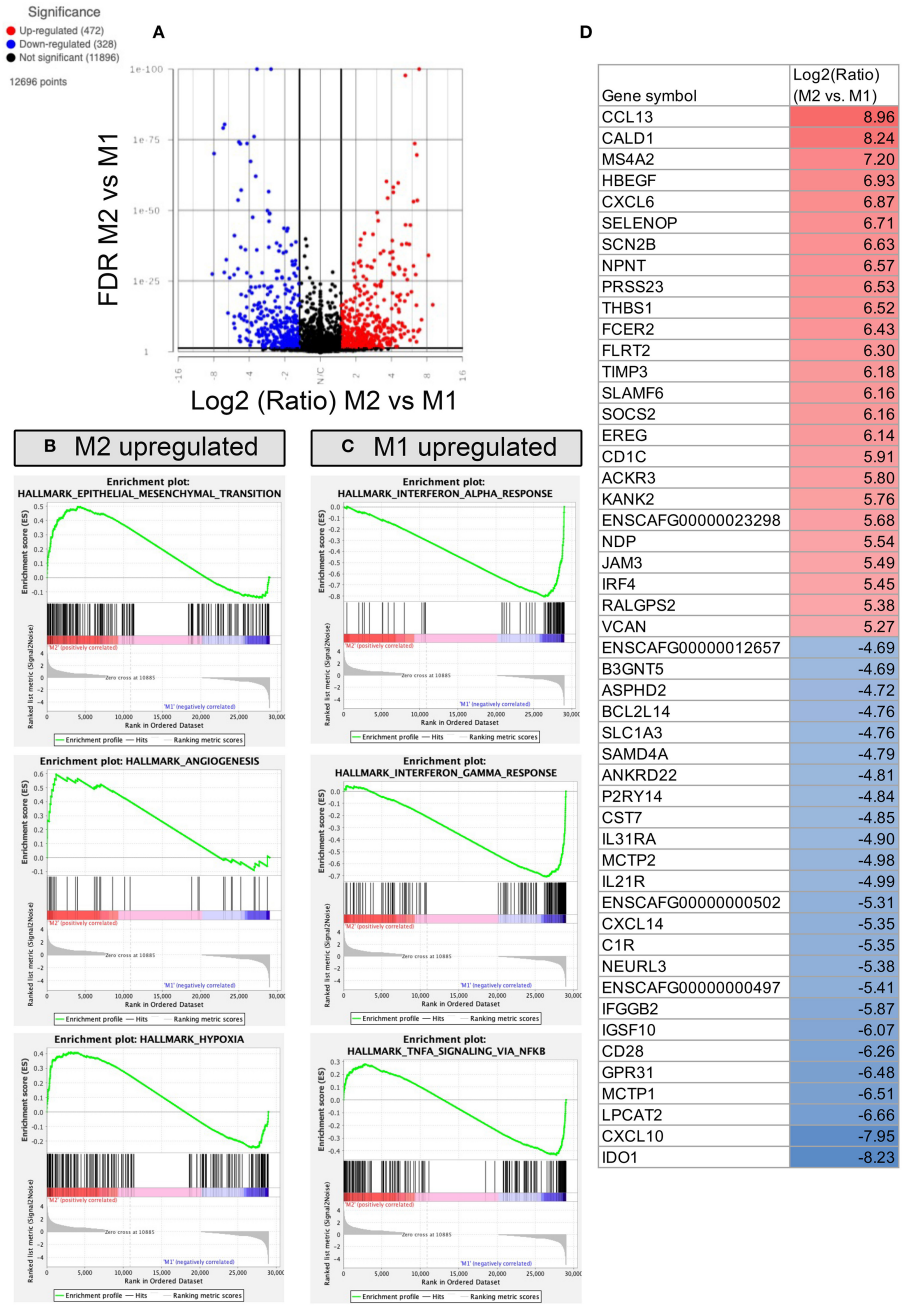
RNA sequencing and transcriptomic analysis of canine polarized macrophages, with comparison of M2 to M1 macrophages. **(A)** A volcano plot of differentially expressed genes, with x-axis cut off (black line) at 1.5 Log2 fold change, y-axis cut off at FDR (false discovery rate) 0.05. Upregulated genes in red, downregulated genes in blue. **(B)** GSEA Hallmarks pathway, using normalized counts generated from transcriptomes of M2 and M1 macrophages. The top 3 significantly upregulated pathways in M2 (*P* < 0.05) are displayed as GSEA plots. **(C)** GSEA top 3 pathways enriched in M1 macrophages, with similar figures as for B. **(D)** Table depicting the top 25 most upregulated and downregulated genes comparing M2 vs. M1 polarized macrophages shown in Log2 fold change, with highest fold change upregulated genes in red, and lowest fold change downregulated genes depicted in blue.

Finally, we used RNA sequencing to elucidate important differences between the two types of macrophage polarization states (Figures 5–7). Macrophage cultures were stimulated with relevant cytokines for 24h, and then mRNA sequencing was performed on the 3 macrophage populations (resting, M1 and M2). Each set of conditions were evaluated independently using macrophage cultures generated from 3 unrelated, healthy dogs. Analysis comparing M1 and M0 macrophages revealed 324 significantly upregulated genes, defined as ≥log2 Fold change, and with an FDR *p*-value cut off of 0.05 (Figure 5A). Significantly upregulated genes in M1 macrophages included IDO1, CXL10, complement C3 (Figure 5C), and others that mapped to pathways including IFN-γ, IFN-α and oxidative phosphorylation (Figure 5B). Other upregulated genes in M1 macrophages included those associated with biological processes such as humoral immune response, oxidative cell stress and apoptotic cell pathways (see Supplementary Table S1 for full gene list).

We also found 538 genes that were significantly downregulated in M1 macrophages compared to M0 macrophages, including genes encoding SOX4, CCL13, TNFSF, CXCR4 (Figure 5D), as well as genes that mapped to epithelial to mesenchymal transition pathways and TGF–β and Hedgehog signaling (Figure 5C). Biological processes downregulated in M1 macrophages included inflammasome signaling, IL-13 production, fluid transport and chemokine production (see Supplementary material S1 for full gene list).

Comparison of anti-inflammatory M2 macrophages to resting M0 macrophages revealed fewer changes in gene expression and identified 161 genes significantly upregulated and 129 genes whose expression was significantly downregulated (Figure 6A). Notable genes that were upregulated included TIMP3, CD209, MPO, IL13RA2 (Figure 6C), and genes which mapped to pathways of oxidative phosphorylation, MTORC1 signaling, Myc targets, and IFN-γ responses (Figure 6B). Biological processes upregulated include negative regulation of exocytosis, chemotaxis, cell adhesion and vesicle targeting (see Supplementary material S1 for full gene list). No hallmark pathways were found to be significantly downregulated in M2 macrophages, however biological processes including NF-kappa signaling, glycolytic process, and respiratory burst were downregulated in M2 macrophages, compared to M0 macrophages.

Direct comparison of the transcriptome of M1 and M2 macrophages revealed that there were 472 genes whose expression was significantly upregulated in inflammatory macrophages compared with anti-inflammatory macrophages, whereas there were 328 genes whose expression was significantly downregulated (Figure 7A). Notable changes included genes that were highly upregulated in M2 macrophages included CCL13, CXCL6, THBS1, and TIMP3 (Figure 7D), mapping to pathways of EMT transition, angiogenesis and hypoxia (Figure 7B). Whereas M1 upregulated genes included IDO1, CXCL10, IL21R, and IL31RA (Figure 7C), many of which overlapped in the comparison of M1 vs. M0 macrophages. Biological processes upregulated in inflammatory M1 macrophages include negative regulation of macrophage activation, defense response to virus, complement activation, response to IFN-β and lymphocyte co-stimulation (see Supplementary material S1 for full gene list). Biological processes upregulated in M2 macrophages compared to M1 macrophages included positive regulation of myeloid leukocyte cytokine production involved in immune response. M1 upregulated biological processes were more numerous and examples included negative regulation of macrophage activation, type 2 immune response, antigen processing and presentation, response to interferon beta, and negative regulation of innate immune response.

### Identification of gene sets used to differentiate macrophage polarization states

**Figure 8:**
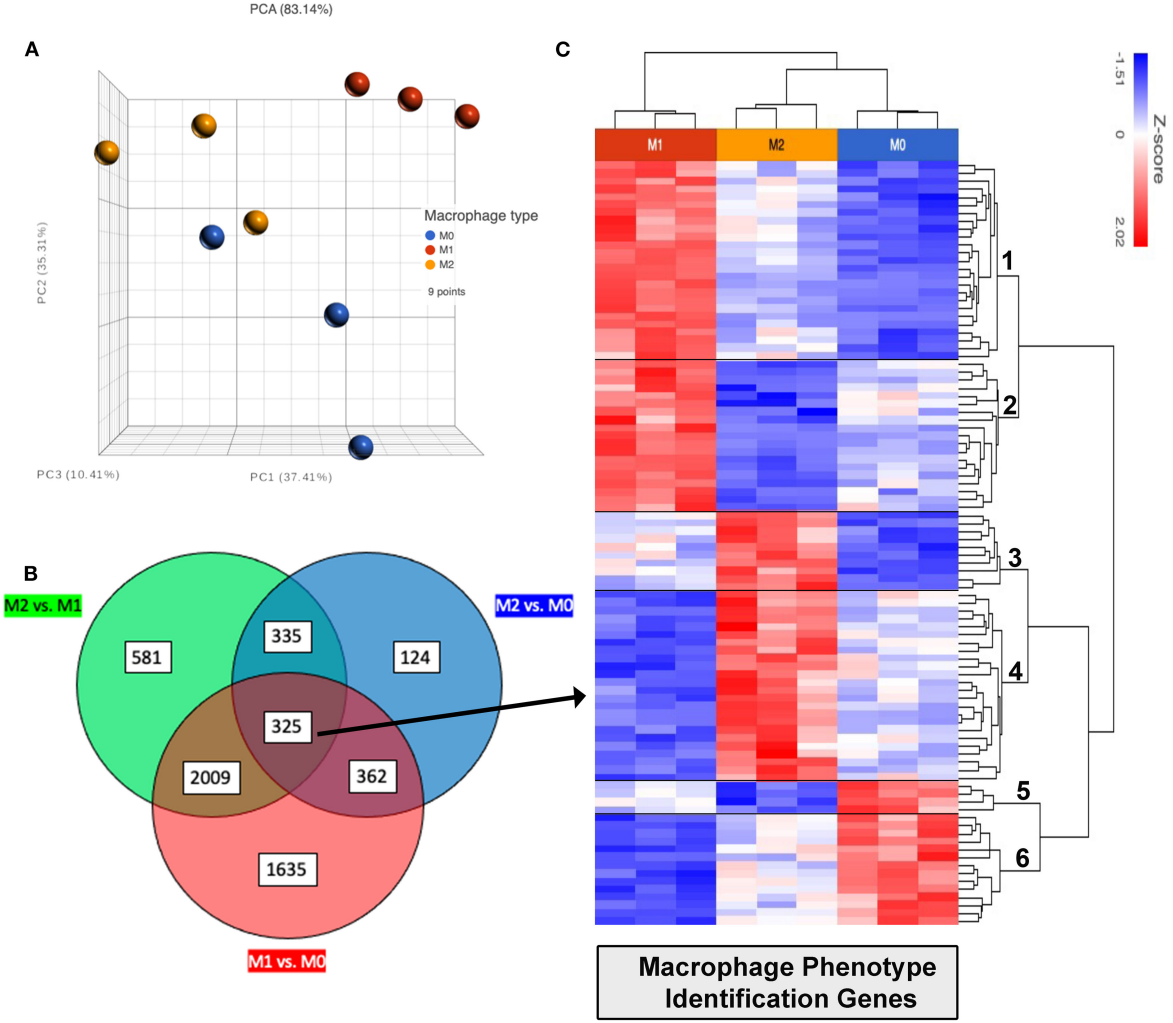
Identification of differentially expressed genes (DEG) related to macrophage activation status. **(A)** PCA (principal component analysis) plot showing distances by variance between biological replicates M0 shown in blue, M1 in red and M2 in yellow. **(B)** Venn diagram showing commonalities of all genes using differentially expressed genes found in comparative analysis shown in Figures 5–7, with FDR ≤ 0.05. Green circle represents total FDR ≤ 0.05 genes when comparing M2 vs. M1. Red circle showing all genes FDR ≤ 0.05 comparing M1 vs. M0. And blue circle showing all genes FDR ≤ 0.05 when comparing M2 vs. M0. Total of 325 genes were found to be shared differentially expressed with FDR ≤ 0.05 when comparing all phenotypes (center circle overlap). **(C)** 325 shared DEGs filtered for normalized count ≥50, 96 genes remaining were then plotted as heat map for cluster analysis, clusters labeled on row dendrogram (right side).

One of the major goals of this study was to identify a gene signature that could be used to define macrophage phenotype in a variety of canine tissues and tumors. A Venn diagram was constructed using the previously mentioned gene lists by filtering for a FDR adjusted *p*-value of 0.05, and including all DEGs with fold change ≥1 or ≤ -1; using (goodcalculators.com/venn-diagram-maker/). In total 325 genes were found in all 3 comparisons (Figure 8B). These genes were then filtered for a normalized count of ≥50, which resulted in a list of 96 genes (Figure 8C) that have high relative deviation between M0, M1 and also M2 macrophages (see Supplementary material S2 for complete list of genes in each cluster), which can be used to differentiate between polarization states. The resulting 96 genes are part of biological processes such as IFN-γ signaling, apoptotic process, immune response, signal transduction, cell migration, and GTPase activity.

Unsupervised hierarchical clustering was used to further divide these 96 genes into related clusters wherein the analysis revealed 6 clusters. Cluster 1 contained 25 genes which had high expression in M1, low expression in M0, and intermediate expression levels in M2 macrophages; including TOP1, RIC1, and JACK2 mapping to DNA replication, protein transport pathways. Cluster 2 contained 19 genes also with high expression in M1, lowest expression in M2, and intermediate or variable expression in M0 macrophages. Genes in cluster 2 include PRNP and FGD and map to molecular categories including catalytic and transcription regulator activity. Cluster 3 contained 10 genes with high expression in M2 and low expression in M1 macrophages, also mapping to catalytic activity. Cluster 4 contained 24 genes with high expression in M2, low in M1, and lower expression in M0 macrophages. These genes also control binding and catalytic activity, as well as transcription regulation and molecule transduction. Within Cluster 4 several genes showed >2 fold upregulation in M2 compared to M1; such as CD9, A4GALT, CSF2RB, NIBAN1. Cluster 5 was the smallest cluster, containing only 4 genes, LY9, MT2A, APBB1IP and MICAL1. These genes have high expression in M0 and low expression in M2 macrophages. Cluster 6 contained 14 genes and encompassed most of the above biological processes in addition to transporter activity. This cluster has high expression in M0, low expression in M1, and variable comparative expression in M2 macrophages.

## Discussion

In this study of polarized canine macrophages, we defined key phenotypic, functional and transcriptomic signatures of M1 (inflammatory) and M2 (anti-inflammatory) macrophages. Consistent with findings in other species, we identified upregulated expression of iNOS, as well as increased bactericidal activity and NO production as key features associated with M1 macrophages. In contrast, anti-inflammatory M2 macrophages were associated with upregulated expression of CD206, arginase, TGM2, and SOCS1 and increased secretion of MCP-1, IL-8, and IL-10, when compared to M1 macrophages. Although secretion of TNF-α was not significantly different by ELISA, gene expression of TNF and TNFRSF1A were significantly decreased when comparing M2 to M1 macrophages by RNA sequencing. Transcriptome analysis revealed significant upregulation of epithelial-to-mesenchymal, angiogenesis, and hypoxia pathways in anti-inflammatory M2 macrophages, whereas M1 macrophages upregulated inflammatory pathways such as IFN-γ, alpha, IL-6 JAK STAT3, and TNF-α. Taken together, these studies generate a more complete understanding of canine macrophage functional polarization states and add to our current understanding of canine macrophage biology.

Our findings are largely in agreement with those of an earlier study of canine macrophage polarization, which also identified upregulated iNOS expression by inflammatory M1 macrophages, as well as upregulation of CD206 expression by anti-inflammatory M2 macrophages (18). Out of the top 50 candidate genes associated with M1 and M2 macrophages in their study; we were able to match more than half to those also identified by mRNA sequencing as being significantly up or down regulated. Some of the discordance in overall gene expression profiles could be attributed to the different cell culture methods, and to the different transcriptomic analysis platform technologies. Our work therefore extends the earlier studies by now providing a more in-depth analysis of the macrophage transcriptome in M1 and M2 polarized macrophages in dogs. Our findings also are largely in accordance with those obtained from analysis of human *in vitro* macrophage gene expression patterns (1) including M2 upregulated genes (e.g., CXCR4, CD36, MRC1, and CCL13) and M1 upregulated genes (e.g., IL15RA, IL7R, CXCL10, and IL15). When compared to larger human *in vitro* datasets also obtained using mRNA sequencing, we found that canine polarized macrophages also matched transcriptomic patterns seen in human M1 and M2 macrophages (11). Compared to published datasets of single cell sequencing in human M1 or M2 macrophages, STAT3 and STAT1 upregulation in M1 macrophages (7, 15, 36). Future studies are underway in our lab, which use single cell RNA sequencing to better define the heterogeneity of canine macrophages in normal tissues as well as tumors.

The panel of potentially cross-reactive macrophage antibodies evaluated in the current study were selected based on prior studies using human and murine macrophages, where prototypical M1 and M2 markers were identified (9, 13, 16, 21, 31, 37). Our studies confirmed the utility of iNOS expression for M1 macrophages and extended the available markers for M2 macrophages to include TGM2 and SOCS1, in addition to the commonly used arginase and CD206 expression. Furthermore, our results suggest there may be an advantage when combining these antibodies (particularly using the ratio of CD206/iNOS) for enhanced ability to distinguish between macrophage polarization states M0, M1, and M2. The RNA sequencing data is particularly valuable as it provides gene sets that can be used to predict functional states of canine macrophages. Interestingly, arginase expression was not significantly different between M2 and M1 macrophages by flow cytometry, immunofluorescence staining, or RNA sequencing (albeit slightly upregulated). These results are in disparity with previously published reports using murine macrophages, which commonly use the dichotomy of iNOS and arginase to identify M2 vs. M1 macrophages (6). This could be attributed to species differences as noted in a previous canine macrophage phenotyping publication (18), as well as the differences between tissue resident macrophages compared to *in vitro* differentiated macrophages (38).

We also found that macrophages upregulated expression of CD86 following IFN-γ treatment to polarize to M1, but interestingly did not upregulate MHCII or CD40 expression. Unlike the case with cultured macrophages, we have previously found that canine dendritic cells generated *in vitro* using GM-CSF significantly upregulated MHCII and CD40 expression when treated with IFN-γ (28).

Phagocytic and bactericidal ability are also important distinguishing characteristics of polarized macrophages. In response to bacterial infections, inflammatory M1 macrophages are essential early responders to bacterial and fungal infections, and ultimately kill the ingested organisms when activated to the inflammatory state (39). Bactericidal activity in canine macrophages can be induced by other stimuli in addition to IFN-γ and TNF-α, including TLR ligands. For example, we recently demonstrated increased phagocytosis and bacterial killing by canine inflammatory macrophages activated by a liposomal-TLR3 and TLR9 immunotherapeutic (23). Expression of iNOS and production of NO as an antimicrobial effector molecule is highly variable between species, with human macrophages producing minimal NO when activated, whereas murine macrophages produce high levels of NO when activated (40). Our studies reveal that canine macrophages produce significant amounts of NO when activated and suggest an important role for this antimicrobial pathway in dogs. Arginase production was not measured in this study as our immunolabeling experiments found that arginase is not an optimal marker to differentiate canine macrophage phenotypes.

The overall picture that emerges from these *in vitro* studies is that M2 polarized canine macrophages are largely unable to mount effective antimicrobial defenses. In contrast, M1 macrophages were significantly more effective in producing NO, and in mounting bactericidal activity against *S. pseudointermedius*. These studies therefore highlight the differences between M1 and M2 macrophages in dogs, and as in other species, M1 macrophages are effective at killing intracellular pathogens, while M2 macrophages largely lack this function.

In conclusion, we found that canine macrophages were readily polarized *in vitro* to M1 macrophages by culture with IFN-γ and to M2 macrophages by culture with IL-4 and IL-13. The study also identified useful markers for phenotypic assessment of the macrophage polarization states in tissues, including iNOS for M1 macrophages and CD206, TGM2, arginase, and SOCS-1 for M2 macrophages. The ratio of CD206/iNOS expression is also able to distinguish between M1 or M2 polarization. Distinctive cytokine secretion profiles were also identified, including upregulated secretion of IL-8 and MCP-1 and IL-10 for M2 macrophages, and upregulated secretion of TNF-α by M1 macrophages. Transcriptome analysis revealed an extended list of genes that can be utilized to identify macrophage polarization states in canine cells and also confirmed similarities in gene expression patterns to *in vitro* human macrophage polarization studies. Using overlapping genes by comparing DEGs between macrophage phenotypes, 6 clusters of genes were identified to be suitable for differentiation between activation states. Taken together, these studies provide new insights into canine macrophage biology and identify both similarities and unique features of dog macrophages relative to human macrophages.

## Data availability statement

The datasets presented in this study can be found in online repositories. The names of the repository/repositories and accession number(s) can be found below: https://www.ncbi.nlm.nih.gov/geo/, GSE207661.

## Ethics statement

The animal study and use of blood samples was reviewed and approved by the Colorado State University Institutional Animal Care and Use Committee.

## Author contributions

LC, WW, SS, and SD: collection and assembly of data, conception, design, and manuscript writing and revision, data analysis and interpretation. DA: collection of data and manuscript revision. All authors contributed to the article and approved the submitted version.

## Funding

This work was supported by PHS funding (1 U01 CA224182-01) and the Charles Shipley Family Foundation. Kasetsart Veterinary Development Fund of Thailand provided funding for SS. DA funded by T32 OD012201.

## Conflict of interest

The authors declare that the research was conducted in the absence of any commercial or financial relationships that could be construed as a potential conflict of interest.

## Publisher's note

All claims expressed in this article are solely those of the authors and do not necessarily represent those of their affiliated organizations, or those of the publisher, the editors and the reviewers. Any product that may be evaluated in this article, or claim that may be made by its manufacturer, is not guaranteed or endorsed by the publisher.

## Supplementary material

Supplemental Figure S1 Representative images of immunofluorescence staining of matched isotype controls for primary antibodies iNOS, CD206, TGM2, Arginase, and SOCS1. Negative staining used for baseline fluorescence thresholding in MFI and percent positive calculations as described in methods “*Macrophage immunocytochemistry”*.

Supplemental Table S1 Full protein coding gene list of RNA sequencing results; table includes DESEQ comparison results between M0, M1, and M2 macrophages with log2 fold change, FDR and *p* values for each comparison.

Supplemental Table S2 Gene list of differentially expressed clusters described in Figure 8. For each unique cluster, *p*-value, FDR adjusted *p*-value and fold changes are listed for M0, M1, and M2 comparisons. Additional info for each cluster 1 through 6 contains full gene description, protein family, and biological processes associated with each gene.
