# Supplementary figures and images for: Canine polarized macrophages express distinct functional and transcriptomic profiles
Source: Front Vet Sci. 2022 Nov 1;9:988981. doi: 10.3389/fvets.2022.988981 (PMC9663804; doi:10.3389/fvets.2022.988981)

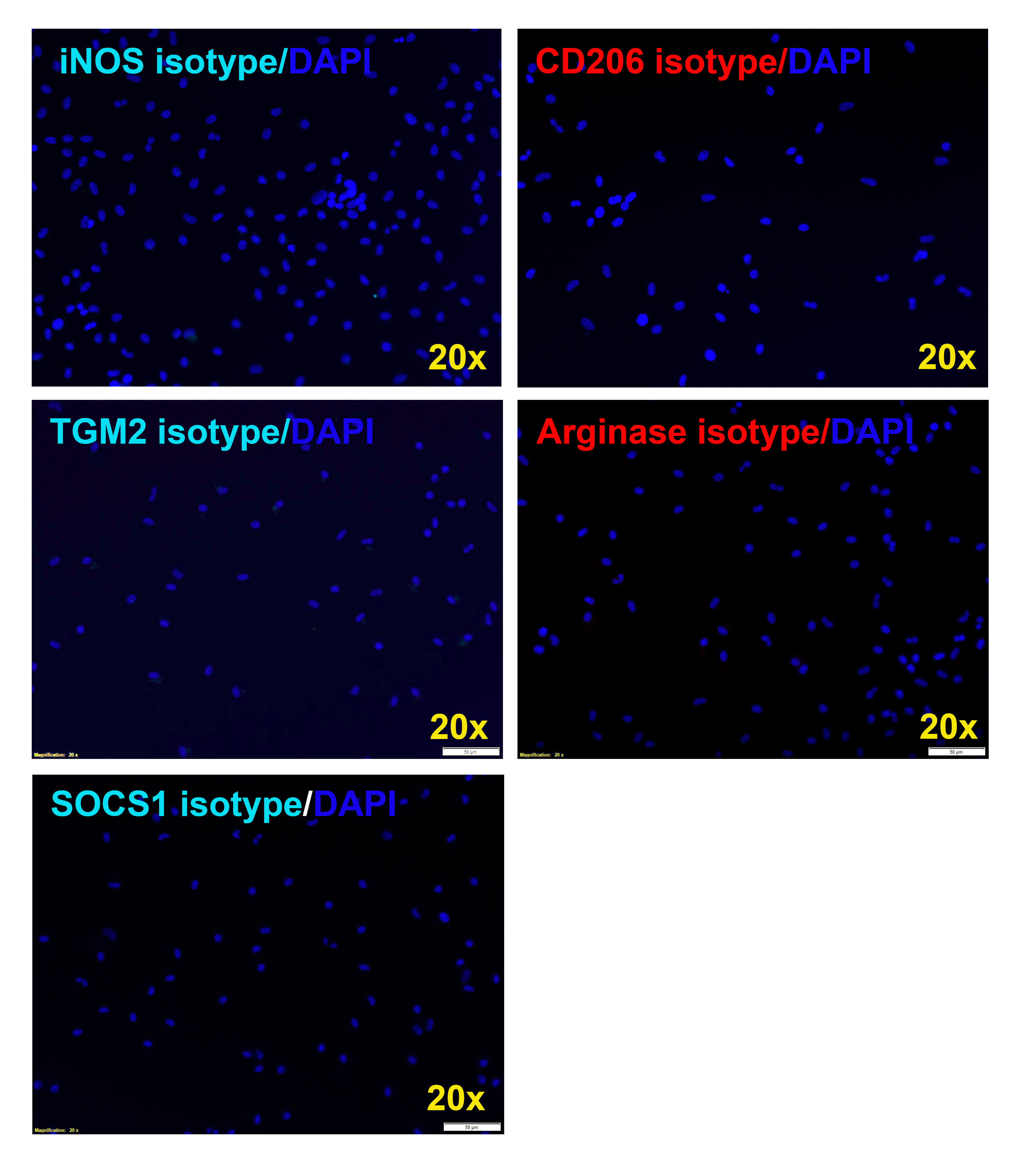

Supplement: Supplemental Figure S1 — Representative images of immunofluorescence staining of matched isotype controls for primary antibodies iNOS, CD206, TGM2, Arginase, and SOCS1. Negative staining used for baseline fluorescence thresholding in MFI and percent positive calculations as described in methods “Macrophage immunocytochemistry”. [file Image_1.TIF]
